# Tightly Controlled Expression of *bHLH142* Is Essential for Timely Tapetal Programmed Cell Death and Pollen Development in Rice

**DOI:** 10.3389/fpls.2017.01258

**Published:** 2017-07-18

**Authors:** Swee-Suak Ko, Min-Jeng Li, Yi-Jyun Lin, Hong-Xian Hsing, Ting-Ting Yang, Tien-Kuan Chen, Chung-Min Jhong, Maurice Sun-Ben Ku

**Affiliations:** ^1^Academia Sinica Biotechnology Center in Southern Taiwan Tainan, Taiwan; ^2^Agricultural Biotechnology Research Center, Academia Sinica Taipei, Taiwan; ^3^Department of Bioagricultural Science, National Chiayi University Chiayi, Taiwan; ^4^School of Biological Sciences, Washington State University, Pullman WA, United States

**Keywords:** bHLH, male sterility, pollen development, rice, ROS scavenging, tapetal PCD

## Abstract

Male sterility is important for hybrid seed production. Pollen development is regulated by a complex network. We previously showed that knockout of *bHLH142* in rice (*Oryza sativa*) causes pollen sterility by interrupting tapetal programmed cell death (PCD) and bHLH142 coordinates with TDR to modulate the expression of *EAT1*. In this study, we demonstrated that overexpression of *bHLH142* (OE142) under the control of the ubiquitin promoter also leads to male sterility in rice by triggering the premature onset of PCD. Protein of bHLH142 was found to accumulate specifically in the OE142 anthers. Overexpression of bHLH142 induced early expression of several key regulatory transcription factors in pollen development. In particular, the upregulation of EAT1 at the early stage of pollen development promoted premature PCD in the OE142 anthers, while its downregulation at the late stage impaired pollen development by suppressing genes involved in pollen wall biosynthesis, ROS scavenging and PCD. Collectively, these events led to male sterility in OE142. Analyses of related mutants further revealed the hierarchy of the pollen development regulatory gene network. Thus, the findings of this study advance our understanding of the central role played by bHLH142 in the regulatory network leading to pollen development in rice and how overexpression of its expression affects pollen development. Exploitation of this novel functionality of *bHLH142* may confer a big advantage to hybrid seed production.

## Introduction

Rice (*Oryza sativa*) is one of the most important staple crops in the world, feeding almost half of the world’s population. Increase in rice production is urgently needed to keep pace with increasing population, especially in the face of drastic global climate change. Hybrid rice is considered the most promising strategy, and can increase rice yield by 15–20% (Khush, 2013). By adopting hybrid technology, many countries have successfully increased per capita rice production (Zhang, 2011). Heterosis in the F1 plants not only increases grain yield but also produces superior phenotypes in comparison with the parents with vigor in growth, good agronomic traits and pest resistance, etc. (Liu et al., 2015). As rice is a self-pollinated crop, adoption of a stable male sterility in the female parent is critical to ensure the purity of F1 seeds. Therefore, a better understanding of the mechanism underlying pollen development is important for developing new genic male sterility lines.

Rice anthers comprise four lobes and each lobe contains four layers of anther walls. The tapetum layer is the innermost layer, providing nutrients and sporopollenin precursors for pollen development. Tapetal programmed cell death (PCD) at the right time is important for normal pollen development. In the anther, PCD is first detectable at meiosis (stage 8, S8), strong PCD signals occur at the young microspore stage (S9) (Li et al., 2006; Zhang and Wilson, 2009), and reduced PCD signals occur at the vacuolated pollen stage (S10) (Hu et al., 2011). Functioning as polar secretory cells, the tapetum undergoes cellular degradation. Tapetal PCD subsequently triggers cytoplasmic shrinkage, breakdown of the nuclear membrane, oligonucleosomal cleavage of DNA, vacuole rupture, and swelling of the endoplasmic reticulum for release of nutrients for pollen development (Papini et al., 1999). Thus, timely tapetal PCD is an essential process for pollen maturation.

Pollen development is controlled by a complex regulatory network. So far, several basic helix-loop-helix (bHLH) transcription factors (TFs) have been identified to play important roles in regulating tapetal PCD and pollen development. The roles of *UDT1* (*bHLH164*) (Jung et al., 2005), *bHLH142* (*TIP2*) (Fu et al., 2014; Ko et al., 2014), *TDR* (*bHLH5*) (Li et al., 2006), and *EAT1* (*DTD1*, *bHLH141*) (Ji et al., 2013; Niu et al., 2013) in rice pollen development have been characterized in the last decade. Similarly, *DYT1*, the homolog gene of *UDT1* in *Arabidopsis* (Zhang et al., 2006) and *AMS* (Sorensen et al., 2003), the homolog of *TDR* in *Arabidopsis*, are functionally conserved in both dicots and monocots. In addition, the *UDT1* homolog in tomato, *ms10^35^* (*Solyc02g079810*), was also recently reported to be involved in pollen development (Jeong et al., 2014). Another TF, GAMYB, is also known to play an important role in anther and aleurone layer development (Kaneko et al., 2004; Tsuji et al., 2006; Aya et al., 2009). According to current understanding of the pollen development regulatory network, UDT1 and GAMYB work in parallel to regulate pollen development and TDR acts downstream of UDT1 and GAMYB (Liu et al., 2010). Our previous study showed that bHLH142 is located downstream of UDT1 and GAMYB and coordinates with TDR through protein–protein interaction to modulate *EAT1* transcriptional activity. In addition, EAT1 interacts with TDR at a similar binding site to bHLH142 (Ko et al., 2014). So far, the biological role of TDR in interacting with EAT1 remains unknown (Ji et al., 2013; Niu et al., 2013; Ko et al., 2014). EAT1 directly regulates tapetal PCD via two aspartic proteases (AP37 and AP25) that activate cell death (Niu et al., 2013). AtTDF1 encodes a R2R3 MYB TF, which functions in callose dissolution (Zhu et al., 2008). Similarly, the rice ortholog, OsTDF1 (MYB35), acts downstream of UDT1 and upstream of TDR, EAT1, OsMYB103, and Persistent Tapetal Cell 1 (PTC1) and it is essential for tapetal PCD (Cai et al., 2015). In addition, PTC1 encodes a PHD-finger TF and controls tapetal PCD and pollen development and acts downstream of GAMYB (Li et al., 2011) and TIP2 (bHLH142) (Fu et al., 2014).

During anther development, ROS acts as a signal to promote tapetal PCD (Hu et al., 2011; Yi et al., 2016). The cellular ROS level is determined by the interplay between ROS-producing and ROS-scavenging mechanisms (Gapper and Dolan, 2006; Miller et al., 2008). MADS3, a floral homeostatic C-class gene required for stamen identity, also regulates ROS scavenging during rice anther development. MADS3 has been shown to modulate ROS levels through positive transcriptional regulation of the promoter of metallothionein gene *MT-1-4b* (Hu et al., 2011). The anthers of *mads3* mutant showed a strong ROS signal and a defect in pollen fertility (Hu et al., 2011). On the other hand, Defective Tapetum Cell Death 1 (DTC1) encodes a protein that contains a development and cell death (DCD) domain and KELCH repeats and acts as a key regulator of tapetum PCD by inhibiting ROS-scavenging activity through its interaction with metallothionein protein MT2b (Yi et al., 2016). Both MT-1-4b and MT2b act as ROS scavengers. Decreased expression of *MT2b* or *MT-1-4b* reduces scavenging activity and causes the accumulation of ROS molecules in rice roots (Steffens and Sauter, 2009) and anthers (Hu et al., 2011). Therefore, a timely buildup of proper ROS level to trigger tapetal PCD during pollen development is vital.

The pollen wall is composed of three layers: pollen coat, outer exine layer, and inner intine layer (Zhang et al., 2016). Biosynthesis, secretion, and translocation of sporopollenin precursors are essential for pollen wall development. Synthesis of sporopollenin precursors is conducted in the tapetum, and *ACOS5*, *CYP703A*, *CYP704B*, *MS2*, etc., play major roles in this process. Ubish bodies transport tapetum-derived sporopollenin precursors to developing exine. Lipidic pollen exine is made of sporopollenin that is derived from the polymerization of fatty acid metabolites and phenolic acid (Ariizumi and Toriyama, 2011). In addition, MYB80/MYB103/MS188 is required for anther development in both *Arabidopsis* and rice (Higginson et al., 2003; Zhang et al., 2007). A recent study further demonstrated that AMS protein interacts with MS188 (MYB80) to modulate promoter of *CYP703A2*, which is involved in pollen wall development (Xiong et al., 2016). Male Sterility1 (MS1), a homeodomain (PHD) finger motif TF, regulates biosynthesis and secretion of pollen wall components in *Arabidopsis* (Wilson et al., 2001; Ito and Shinozaki, 2002; Yang et al., 2007). A subsequent study found that *PTC1*, a *MS1* homolog in rice, is also essential for tapetal PCD and pollen development in rice (Li et al., 2011). Several genes associated with rice pollen wall development have been identified by microarray analysis; these include *Cys protease* (*CP1*; Lee et al., 2004), a fatty acyl-CoA reductase homologous to *Arabidopsis MS2* (Aarts et al., 1997), lipid transfer proteins such as *C4* (Tsuchiya et al., 1994), *C6* (Zhang et al., 2010), *YY1*, BURP domain-containing proteins (*RA8* and *OsRAFTIN*; Jeon et al., 1999), and a P450 family member *CYP704B2* (Li et al., 2010). They were all downregulated in the anther of the rice *ptc1* mutant (Li et al., 2011). Moreover, mutagenesis studies suggest that *CYP703A2* (Yang et al., 2014) and *CYP704B2* (Li et al., 2010) are essential for pollen development, and their knockout lines exhibited impaired pollen development. *MS2* is essential for pollen wall biosynthesis by mediating the production of the conserved plastidial pathway for the production of fatty alcohols that are essential for pollen wall biosynthesis (Chen et al., 2011; Shi et al., 2011). Clearly, interruption of the functions of these genes resulted in abnormal pollen development.

In rice, *bHLH142* is specifically expressed in the anther and regulates tapetal PCD and pollen development, and knockout of *bHLH142* causes pollen sterility (Fu et al., 2014; Ko et al., 2014). To gain more insight into its functionality, in this study we generated transgenic lines overexpressing *bHLH142* under the control of the maize ubiquitin promoter. A very recent study suggested that constitutive overexpression of *bHLH142* defects pollen development and anther dehiscence in rice (Ranjan et al., 2017). In the present study, we demonstrated that constitutive overexpression of *bHLH142* also leads to male sterility in rice by triggering premature tapetal PCD via altered expression of *EAT1*. Moreover, we conducted more detailed molecular, histological and biochemical analyses on the effects of overexpressing bHLH142 on rice pollen development and used related mutants to reveal the hierarchy of these pollen development regulatory genes. Thus, our findings advance our understanding of the central role played by bHLH142 in the regulatory network leading to pollen development in rice. Based on our discoveries, we also proposed a new method to generate genic male sterility in rice and possibly in other cereal crops too for agricultural application.

## Materials and Methods

### Constructs

The *bHLH142* (Os01g0293100) full-length cDNA was PCR amplified using primers S80qPCR-F3_BamHI and S80FLcds-R2_BamHI (see Supplementary Table S1) and the product is a 1373 bp BamHI fragment. This fragment was then digested with BamHI and ligated into pCAMBIA1390 backbone containing the maize ubiquitin promoter. Expression of the selection marker *HptII* gene that encodes hygromycin phosphotransferase was driven by cauliflower mosaic virus (CaMV) 35S promoter (Supplementary Figure S1E). Another vector harboring fused bHLH142 and eGFP (Ubi::bHLH142-eGFP) was constructed to detect the tissue specificity of bHLH142 protein using eGFP. All constructs were confirmed by sequencing. The plasmids were separately transformed and selected by antibiotic. *Agrobacterium tumefaciens* strain EHA105 was used for transfection to calli of TNG67 background following the method described previously (Chan et al., 1993).

### Plant Material and Growth Conditions

Transformation of Japonica rice cultivar TNG67 was described previously (Ko et al., 2014). Primary transgenic lines were transplanted into soil and cultivated in the Academia Sinica-BCST greenhouse for genetically modified organisms, in Tainan, Taiwan.

### Histochemical Staining

Transverse paraffin sections of anther were sectioned, deparaffined, rehydrated, and stained for starch with 2% I_2_/KI solution. Sudan Black B (0.3%, w/v; Sigma, Lot#MKBQ9075V) prepared in 70% ethyl alcohol was used to stain lipids, as described previously (Oliveira, 2015).

### TUNEL Assay

To investigate the breakdown of tapetal PCD, TUNEL assay was performed using the DeadEnd Fluorometric TUNEL system (Promega) as described previously (Ko et al., 2014). Anther development stages from microspore mother cell (MMC, S7) to vacuolated pollen (S10) were collected.

### ROS Staining and Activity Assay

Anthers of Wt and OE142 line #96 at various developmental stages were collected. Superoxide anion was quantified using a water-soluble tetrazolium salt reagent WST-1: Na,2-[4-iodophenyl]-3-[4-nitrophenyl]-5-[2,4-disulfophenyl]-2H-tetrazolium), as described previously (Yi et al., 2016).

### RNA Isolation and qRT-PCR Analyses

Rice (*Oryza sativa*) spikelets at different developmental stages, sporogenous cell (SC, S6), MMC (S7), meiosis (Mei, S8), young microspore (YM, S9), vacuolated pollen (VP, S10), pollen mitotic (PM, S11), and mature pollen at 1 day before anthesis (MP, S12), were collected for total RNA isolation, using LiCl2 method (Wang and Vodkin, 1994). One microgram of RNA was used to synthesize the oligo(dT) primed first-strand cDNA using the M-MLV reverse transcriptase cDNA synthesis kit (Promega). One microliter of the reverse transcription products was used as a template in the qRT-PCR reactions following previous protocols (Ko et al., 2014). *Ubiquitin-like 5* (*UBQ5*, Os01g0328400) was used as an internal control for normalization of expression levels.

### Protein Gel Blot Analysis

Total protein was extracted from newly matured leaves with Culture Cell Lysis Reagent (CCLR) buffer (100 mM K_2_HPO_4_, 100 mM KH_2_PO_4_, pH 7.8, containing 1% Triton X-100, 10% glycerol, 1 mM EDTA, and 7 mM 2-mercaptoenthanol). Protein concentration was measured using the Bio-Rad Protein Assay Kit with bovine serum albumin as a standard.

For Western blot analysis, 80 μg total protein from each sample was loaded and separated by SDS-PAGE with a 12% acrylamide gel and transferred onto polyvinylidene fluoride (PVDF) membrane for antibody probing. Antibodies against the rice bHLH142 and EAT1 were produced against the synthetic peptide (CSPTPRSGGGRKRSR) and (CELKILVEQKRHGNN), respectively. The following primary antibodies were used: Anti-bHLH142, rabbit polyclonal antibody (Genscript) at 1:4000 dilution, Anti-EAT1 rabbit polyclonal antibody (Genscript) at 1:2000 dilution, and Anti-eGFP rabbit polyclonal antibody (Yao-Hong Biotechnology, Cat#YH-80005) at 1:10000 dilution. Anti-actin mouse monoclonal antibody (Sigma, A0480) at 1:2500 dilution was used as equal loading control.

### GFP Fluorescence Microscopy

Spikelet of the Wt, Ubi::bHLH142-eGFP and Ubi::GFP transgenic lines at the S9 to S10 stages were used for GFP florescence observation. GFP signal was recorded using a Zeiss LSM710 confocal microscope equipped with a T-PMT under an FITC filter at excitation of 488 nm and emission wavelength of 500–560 nm.

### RNA *In Situ* Hybridization

Anthers of the non-transgenic Wt and OE142 at various developmental stages were collected and prepared in 10 μm thickness paraffin sections. Dig-labeled RNA probes of *bHLH142* and *EAT1* were cloned and prepared in advance. Hybridization protocols were as previously described (Ko et al., 2014).

## Results

### bHLH142 Overexpressing Transgenic Lines Exhibit Male Sterility

**FIGURE 1:**
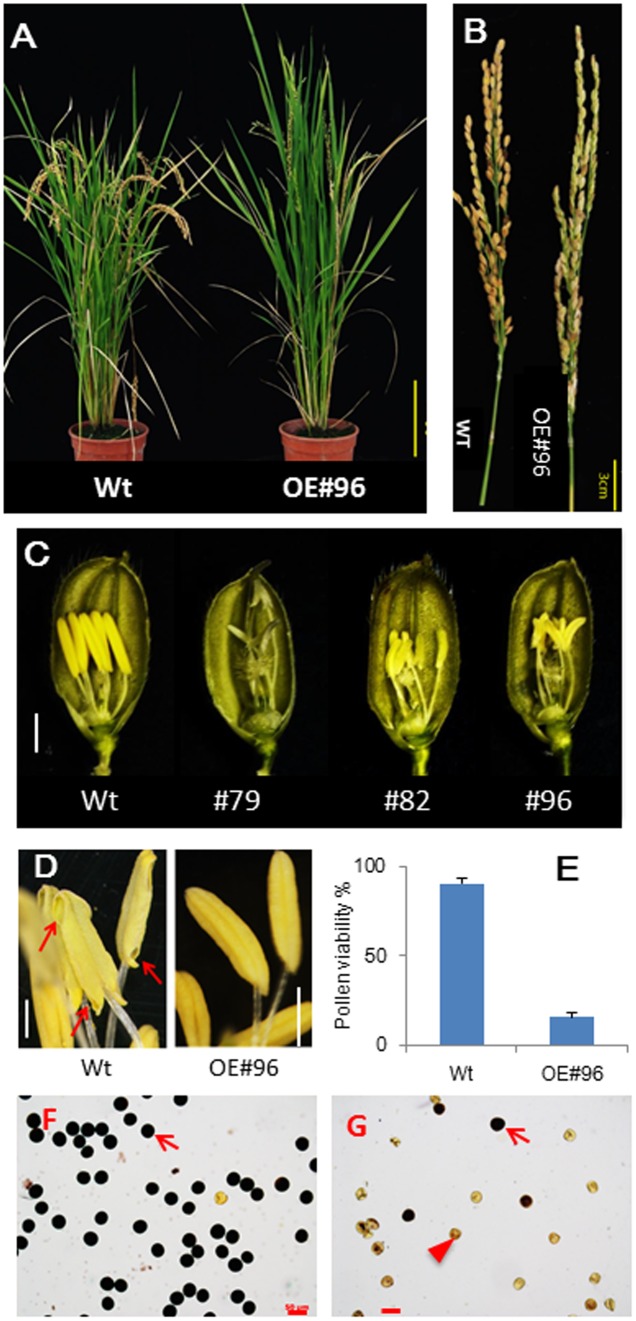
Overexpression of *bHLH142* (OE142) caused male sterility in rice. **(A)** Plant phenotype of wild-type (TNG67, Wt) and OE142 line #96 at seed maturation stage. **(B)** Panicles of Wt and OE142 at seed maturation stage. **(C)** Spikelets of Wt (left) and several OE142 T0 lines at 1 day before anthesis. **(D)** Anthers dehiscence in the Wt but not in OE142 transgenic line. **(E)** Percentage of pollen viability in the Wt and OE142 line #96. **(F)** Staining of pollen grains by 2% I2/KI solution in the Wt. **(G)** Staining of pollen grains by 2% I2/KI solution in OE142 line #96. Error bars indicate the SD of mean from three biological replicates **(E)**. Arrows show the dehisce anther **(D)**. Arrow shows fertile pollen, arrowhead shows infertile pollens **(G)**. Scale bars: 20 cm **(A)**, 3 cm **(B)**, 2 mm **(C)**, 20 μm **(D)**, 50 μm **(F,G)**.

For functional genomics studies, we generated transgenic rice lines overexpressing *bHLH142* under the control of the maize constitutive ubiquitin promoter in the japonica cultivar TNG67 (wild-type, Wt) (**Figure 1A**). More than 15 primary transgenic lines overexpressing *bHLH142* (OE142) were obtained and none of them produced viable seeds at the maturation stage. Genomic PCR confirmed T-DNA insertion in the OE142 lines (Supplementary Figure S1F). With the exception of male sterility, the transgenic plants displayed Wt-like agronomic traits but slightly increased vegetative growth due to lack of grain filling (**Figure 1**). All OE142 transgenic lines produced smaller anthers compared to the Wt (**Figure 1C** and Supplementary Figure S1B). Wt anthers dehisced normally during anthesis but OE142 anthers did not (**Figure 1D**, right panel). Also, Wt exhibited a high percentage of pollen viability and many pollen grains were stained by I_2_/KI but low pollen viability was observed in the pollen of OE142 line (**Figure 1E** and Supplementary Figure S2A). Finally, OE142 plants failed to produce viable seeds (**Figure 1B** and Supplementary Figure S1D).

To elucidate the defect in pollen maturation in OE142, detailed histological assays were carried out. OE142 anther produced less viable pollen grains, as demonstrated by I_2_/KI staining (**Figure 1E** and Supplementary Figure S2A). Moreover, OE142 anther showed very weak Sudan Black staining of lipids compared to the Wt (Supplementary Figure S2B). Histochemical staining analyses suggested that defect in starch and lipid synthesis in the OE142 anthers may be caused by overexpression of *bHLH142*. Transverse section examination showed that OE142 anther entered the meiosis stage and the microspores were released into anther locules (Supplementary Figure S3). However, abnormal anther development in OE142 was observed at the vacuolated pollen stage (S10) where epidermal layer was not thickened. Degeneration of OE142 pollen was observed at the pollen mitotic stage (S11) (Supplementary Figure S3). At the anther maturation stage, Wt showed thickening of endothecial cell layers, ready for dehiscence (Supplementary Figure S2C) but OE142 endothecial cell layers remained thin and no dehiscence took place (Supplementary Figures S2C, S3). Finally, severely degenerated pollen grains were observed in OE142 at the anther maturation stage (Supplementary Figures S2C, S3).

### OE142 Shows Premature Onset of Tapetal PCD

**FIGURE 2:**
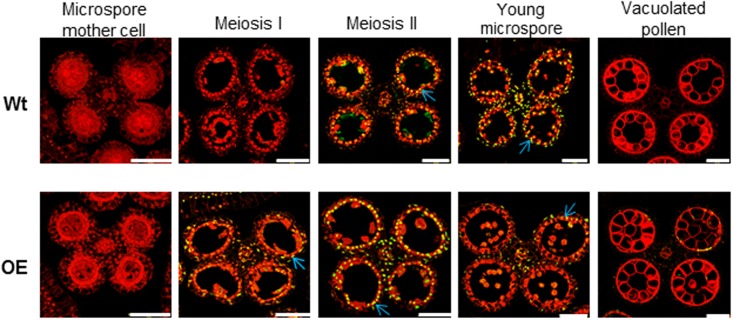
TUNEL assay showing premature onset of tapetal programmed cell death in the OE142 anther. DNA fragmentation signals (yellow fluorescence) started at the meiosis II stage (S8b) and exhibited obvious positive TUNEL signals at the young microspore stage (S9) in the Wt **(Upper)**. Early DNA fragmentation signals were observed in the tapetum of OE142 at meiosis I (S8a), and increased TUNEL positive signals occurred at the meiosis II (S8b) stage **(Lower)**. The red signal is propidium iodide staining, and the yellow fluorescence is the merged signal from TUNEL (green) and propidium iodide staining (red). Arrows indicate signal of tapetal PCD. Scale bars: 50 μm.

As defect in pollen development was observed in OE142 anther (**Figure 1** and Supplementary Figures S1–S3), we suspected that overexpression of *bHLH142* might have altered tapetal PCD, which is responsible for tapetum degeneration during maturation (Papini et al., 1999). Therefore, TUNEL assay was performed to detect DNA fragmentation in the anthers of OE142 line in comparison to Wt. As shown in **Figure 2**, Wt exhibited a normal tapetal PCD signal starting from meiosis-II stage (S8b), which was increased at the young microspore stage (S9). However, premature onset of tapetal PCD was clearly observed in the OE142 anthers, which started at stage S8a with the highest DNA fragmentation signal occurring at S8b, but reduced PCD at S9 (**Figure 2**). The corresponding TUNEL differential image contrast (DIC) images showing the anatomy of anther are presented in Supplementary Figure S4. These data indicate that overexpression of *bHLH142* triggered premature onset of tapetal PCD at S8a. However, OE142 lost timely tapetal PCD at S9 that is critical for releasing nutrients to nurture microspore development, leading to defected pollen maturation.

### Molecular Changes in OE142

**FIGURE 3:**
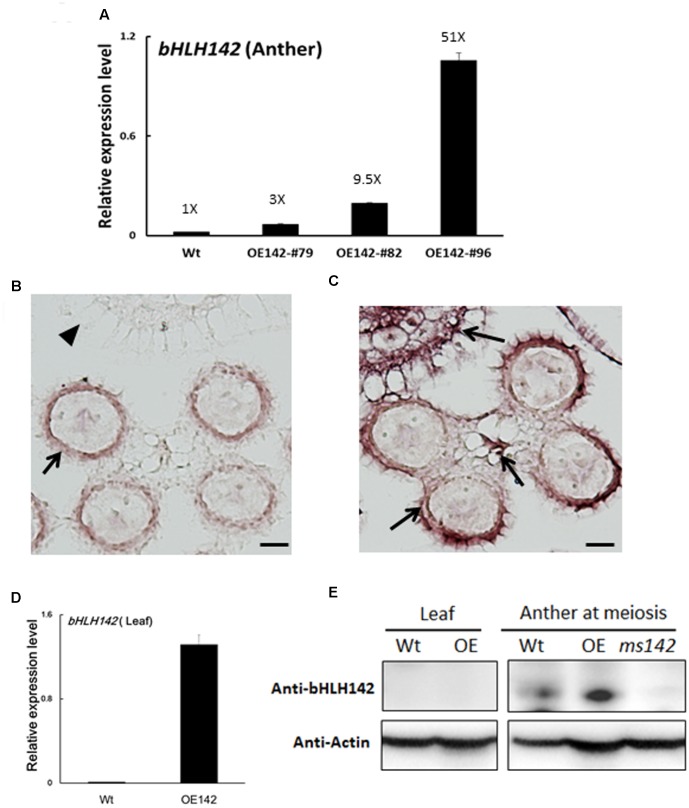
Expression patterns of bHLH142 mRNA and protein in the OE142 transgenic line. **(A)** qRT-PCR showed upregulation of *bHLH142* transcript in the anther of OE lines at young microspore stage. **(B)** RNA ISH of *bHLH142* antisense probe hybridization in the Wt anther at meiosis stage (S8). **(C)** RNA ISH of *bHLH142* antisense probe hybridization in OE142 anther at meiosis stage (S8). **(D)** Overexpression of *bHLH142* significantly increased *bHLH142* transcripts in the leaves of OE142 as analyzed by qRT-PCR. **(E)** Protein of bHLH142 was not expressed in the leaves of OE142 as analyzed by Western blot analysis. It was specifically expressed in the anthers. KO mutant, *ms142*, was included as a negative control for Western blotting of bHLH142. Error bars indicate SD of mean from three technical replicates **(A,D)**. Arrows indicated ISH positive signals in the anther walls **(B)**; ISH positive signals in the hulls, vascular bundle, and anther walls of OE142 **(C)**. The arrowhead indicates no ISH signal in the hull of Wt **(B)**. Scale bars: 20 μm **(B,C)**.

Three OE142 lines with varying expression levels (#79, #82, and #96) were propagated vegetatively for further molecular studies (**Figure 1C**). Real time PCR analysis further showed that the *bHLH142* transcript was 3.3-, 9.5-, and 51.7-fold higher in the anthers of these respective lines, compared to that of Wt anther (**Figure 3A**). Irrespective of *bHLH142* transcript abundance, all three OE lines failed to produce fertile grains as a result of defect in pollen viability. This result implies that a proper expression level of *bHLH142* at the right stage is critical for maintaining normal pollen development in rice. Our result indicated that tightly controlled homeostasis of bHLH142 is essential for the regulatory cascade of pollen development. As OE142 line #96 expressed most abundant *bHLH142* mRNA it was used for further molecular characterization, unless otherwise indicated.

### bHLH142 Protein Is Specifically Expressed in the Anther

**FIGURE 4:**
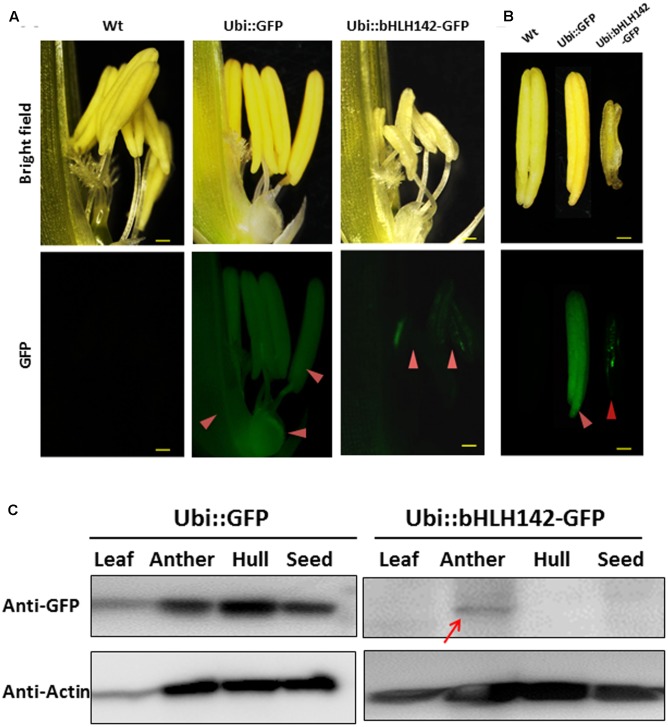
Protein of bHLH142 is specifically expressed in the anther. GFP signals observed under fluorescence microcopy for spikelet **(A)** and anthers **(B)**. Upper panel shows bright field and lower panel shows GFP signals. Arrowheads show GFP signals in the spikelets and anthers of transgenic lines. Scale bars: 50 μm **(A)**, 20 μm **(B)**. **(C)** Western blotting using anti-eGFP antibody indicated bHLH142 protein is tissue specifically expressed in the anther of Ubi::bHLH142-GFP plant only. Transgenic rice overexpressing GFP as driven by ubiquitin promoter was included as a control. Anthers at vacuolated pollen stage were collected for protein isolation.

Previous RNA *in situ* hybridization (ISH) analysis indicated that *bHLH142* is tissue specifically expressed in the anthers of Wt at S7 to S9 but not in the leaf (Fu et al., 2014; Ko et al., 2014). Our RNA ISH data indicated that *bHLH142* transcript was localized specifically in the tapetum, middle layer, and meiocytes of the Wt (**Figure 3B**). However, in OE142 transgenic line, *bHLH142* transcript was detected in both anther (**Figure 3C**) and leaf (**Figure 3D**). Moreover, RNA ISH analysis further demonstrated that *bHLH142* transcript was constitutively expressed in the hulls, anther walls, vascular bundle, and meiocytes of OE142 (**Figure 3C**). Surprisingly, Western blot analysis using anti-bHLH142 antibody showed that bHLH142 protein is only present in the anther but absent in the leaf of OE142 (**Figure 3E**). In addition, using Ubi::bHLH142-GFP transgenic plants generated in this study we demonstrated that GFP fluorescent signal was detected only in the anther but not in the hull of the transgenic line (**Figure 4**). Consistently, Western blot analysis of various tissues from Ubi::GFP and Ubi::bHLH142-GFP plants further demonstrated that GFP protein is only present in the anther but not in the leaf, hull or seed of Ubi::bHLH142-GFP transgenic line (**Figure 4C**, right panel). The transgenic line overexpressing Ubi::GFP served as a good positive control showing constitutive expression of GFP protein in all tested organs (**Figure 4C**, left panel). These results suggest that both Ubi::bHLH142 (OE142) and Ubi::bHLH142-GFP constructs drove the expression of bHLH142 protein specifically in the anther. Clearly, bHLH142 protein is expressed in an anther-specific manner in OE142. Moreover, both Ubi::bHLH142 (OE142) and Ubi::bHLH142-GFP transgenic lines caused a similar male sterility phenotype, presumably due to the overexpression of bHLH142.

### Overexpression of bHLH142 Alters Transcriptional Regulation of Several Known Genes Related to Pollen Development

**FIGURE 5:**
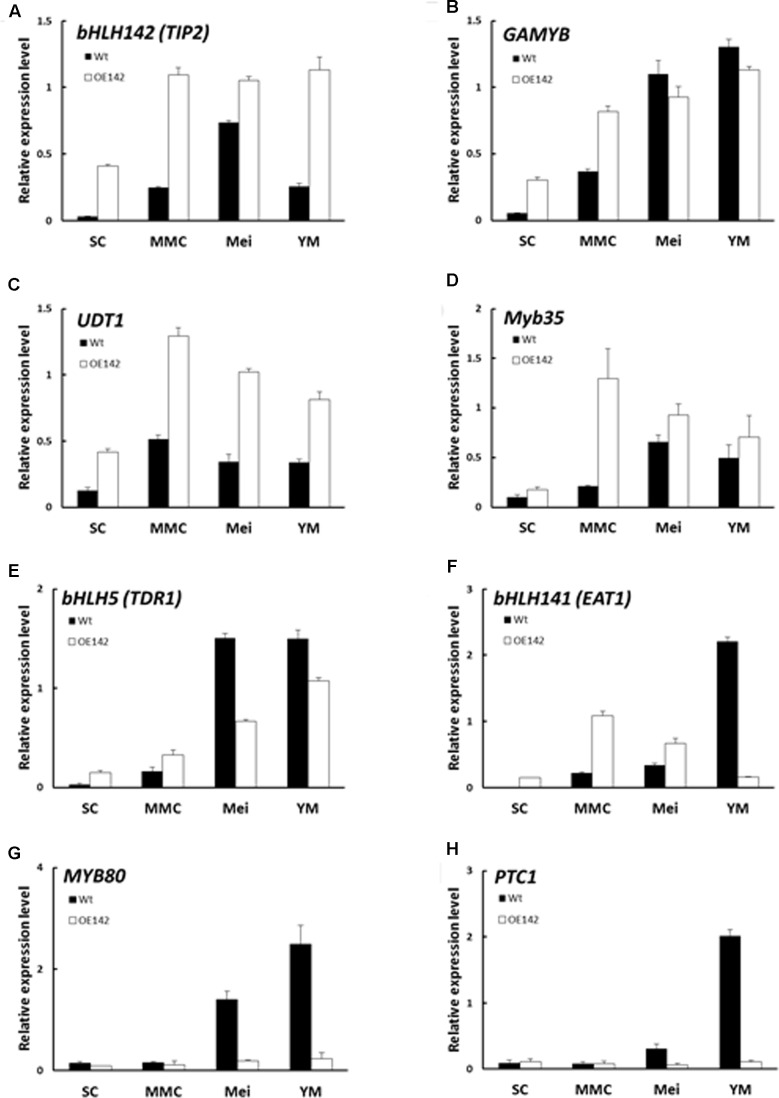
Overexpression of *bHLH142* altered expression in some transcription factors involved in pollen development. Comparison of Wt and OE142 using qRT-PCR to analyze gene expression patterns of *bHLH142 (TIP2)* **(A)**, *GAMYB* **(B)**, *UDT1* **(C)**, *Myb35* **(D)**, *bHLH5 (TDR)* **(E)**, *bHLH141 (EAT1)* **(F)**, *MYB80* **(G)**, *PTC1* **(H)**. SC, sporogenous cell, S6; MMC, microspore mother cell, S7; Mei, meiosis, S8; YM, young microspore, S9. Error bars indicate the SD of mean from three technical replicates.

To understand whether the pollen development regulatory network in OE142 was altered, qRT-PCR analysis of several of the known regulatory TFs that are involved in pollen development was carried out. As the expression of *bHLH142* in the OE142 lines was driven by the strong constitutive ubiquitin promoter, its mRNA expression in the OE142 lines was consistently upregulated throughout all stages of anther development (**Figure 5A**). Interestingly, the expression of *GAMYB*, *UDT1 (bHLH164)*, and *MYB35* (*TDF1*) was also upregulated in the OE142 anthers (**Figure 5**). *TDR* (*bHLH5*) was upregulated at the early stage but then downregulated at the meiosis stage (S8) in OE142 (**Figure 5E**). Similarly, *EAT1* was upregulated at the early meiosis stages from S6 to S8 but strongly suppressed after reaching the young microspore stage (S9) in the anthers of OE142 (**Figure 5F**). In addition, the expression of *MYB80* was found downregulated at meiosis onward (**Figure 5G**). Similar to *MYB80*, *PTC1*, a key regulator of tapetal PCD and pollen wall biosynthesis (Li et al., 2011), declined significantly to a negligible amount at S9 in the OE142 anthers (**Figure 5H**). Clearly, constitutively overexpressing *bHLH142* alters the expression of the key regulatory TFs associated with pollen development.

### Overexpression of bHLH142 Downregulates PCD Related Functional Genes

**FIGURE 6:**
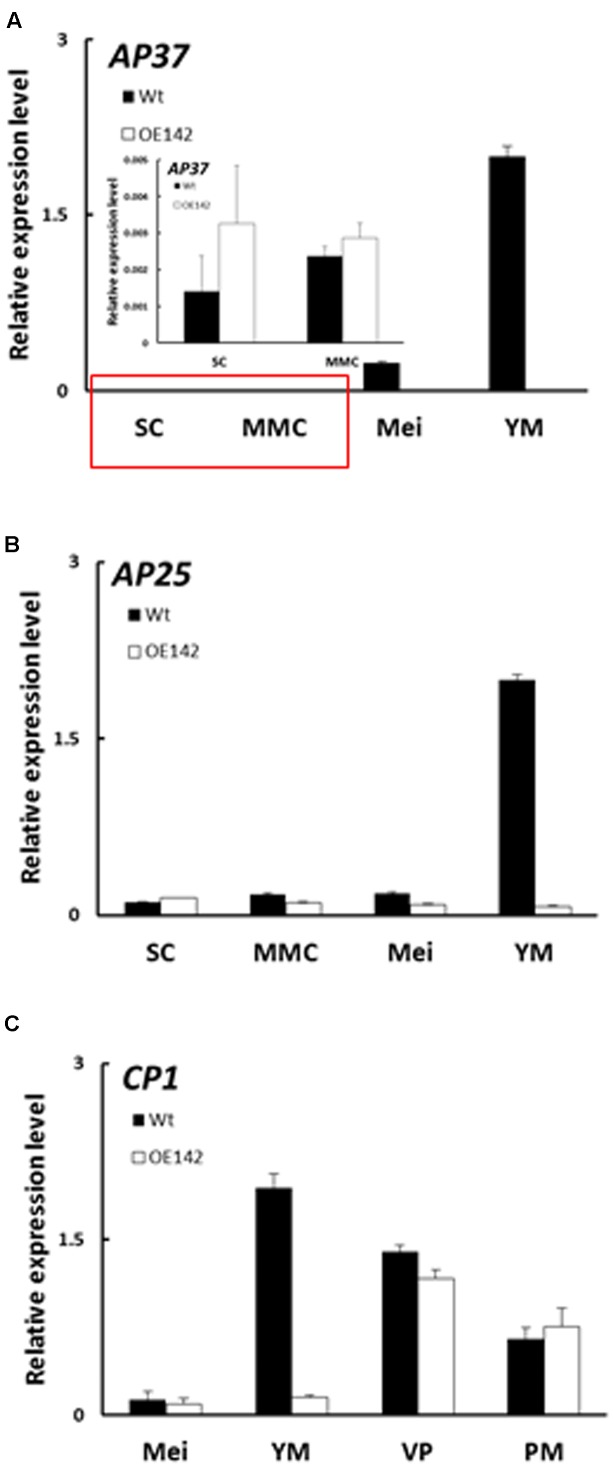
Overexpression of *bHLH142* altered expression patterns in tapetal PCD associated genes. **(A)** Gene expression of *AP37*. Red area is magnification. **(B)** Gene expression of *AP25*. **(C)** Gene expression of *CP1*. Abbreviations are as described in the legend for **Figure 5**.

EAT1 is known to trigger tapetal PCD by regulating the expression of two *Aspartic Proteases* (*AP37*, *AP25*) at the young microspore stage (Niu et al., 2013). In OE142 anthers, *EAT1* was significantly downregulated at S9 (**Figure 5F**). TUNEL assay indicated premature onset of tapetal PCD in OE142 anthers (**Figure 2**), which was correlated with the upregulation of *EAT1* before the meiosis stage (**Figure 5F**). Slightly higher expression of *AP37* and *AP25* at the early stages of OE142 anther development was observed (**Figure 6**). Normally, Wt rice exhibits the strongest expression of *EAT1*, *AP37*, *AP25*, and *CP1* at S9 to turn on timely tapetal PCD (**Figures 5F**, **6**). Thus, the negligible expression levels of these marker genes associated with PCD at S9 in the anthers of OE142 further supports the notion that decreased expression of these tapetal functional genes might disrupt timely tapetal PCD (**Figure 2**). Consistently, reduced expression of these two proteases (*AP37* and *AP25*) coincided with the reduction of *EAT1* mRNA in OE142 at S9 (**Figure 5F**). Thus, collectively these results further support the previous finding that EAT1 regulates *AP37* and *AP25* (Niu et al., 2013).

### OE142 Has Altered ROS Metabolism in the Anther

**FIGURE 7:**
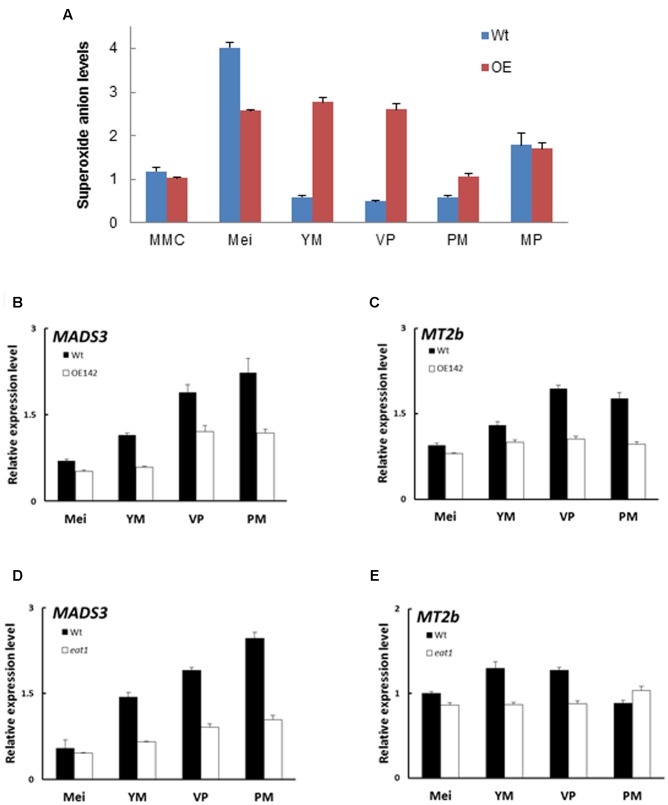
Overexpression of *bHLH142* altered superoxide anion accumulation and the expression of ROS-associated genes. **(A)** Alter superoxide anion levels in the anthers of OE142. **(B)** Comparison of Wt and OE142 using qRT-PCR to analyze gene expression patterns of *MADS3* and *MT2b* **(C)**. **(D,E)** Mutagenesis analysis indicated gene hierarchy of *MADS3* and *MT2b* locate downstream of *EAT1*. Wild-type for *eat1* is in Hitomebore background. Abbreviations are as described in the legend of **Figure 5**.

Timely accumulation of ROS is essential to induce PCD during tapetum degeneration (Hu et al., 2011; Yi et al., 2016). The premature onset of tapetal PCD as shown in **Figure 2** prompted us to hypothesize that OE142 may have altered ROS metabolism in the anthers. Therefore, quantitative measurement of superoxide ion using WST-1 was performed in the anthers of the Wt and OE142 line at various developmental stages. Normally, ROS accumulates more at S8 to S9 to trigger tapetal PCD (Xie et al., 2014). Our results showed that the Wt anthers accumulated the highest level of superoxide anions during the meiosis stage; however, OE142 had significantly lower level of superoxide anions compared to the Wt. In addition, the anthers of OE142 accumulated more superoxide anions at the later stage of anther development (**Figure 7A**) which might be toxic for OE142 anther development. A previous study suggested that tapetal PCD requires timely and precise control of ROS levels (Xie et al., 2014). We therefore compared the expression of rice ROS scavenging-associated genes in OE142 at various stages of anther development. Our qRT-PCR analysis demonstrated that OE142 significantly reduced the expression of *MADS3* and *MT2b* (**Figures 7B,C**), consistent with the higher ROS accumulation in OE142 as compared to the Wt (**Figure 7A**). Our results support the suggestion that *MADS3* is involved in ROS metabolism in triggering PCD (Hu et al., 2011). Taken together, these results suggest that decreased ROS scavenging activity in OE142 anthers affects ROS metabolism and initiation of synchronized PCD, resulting in defective pollen grains (**Figure 1E**). Based on the fact that *EAT1* expression is downregulated at the S9 stage in OE142 anthers, we contemplated whether *EAT1* may also play an important regulatory role in ROS metabolism. To verify the possible gene hierarchy in this regulatory process, qRT-PCR analyses of the expression of ROS marker genes in *eat1* (Tos17 mutant) anthers were carried out. The results indicated that *MADS3* and *MT2b* were downregulated in *eat1* mutant (**Figures 7D,E**), implying that *MADS3* and *MT2b* genes might be located downstream of the *EAT1* regulatory network. Taken together, these data suggest that overexpression of *bHLH142* causes downregulation of *EAT1* at the late stage of anther development, which in turn alters the expression of ROS scavenging genes with decreased scavenging activity and accumulates ROS molecules, leading to defected male gametophyte development.

### Overexpression of bHLH142 Impairs Sporopollenin Biosynthesis

**FIGURE 8:**
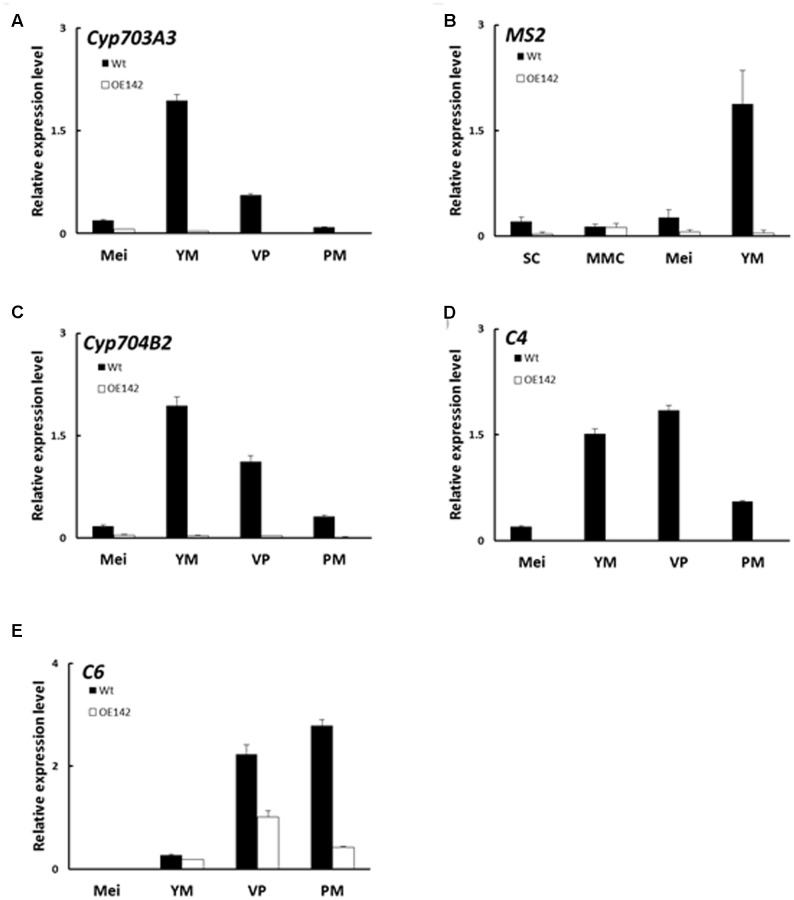
Overexpression of *bHLH142* altered the expression of genes associated with pollen wall biosynthesis. Comparison of Wt and OE142 using qRT-PCR to analyze gene expression patterns of *Cyp703A3* **(A)**, *MS2* **(B)**, *Cyp704B2* **(C)**, *C4* **(D)**, and *C6* **(E)**. Abbreviations are as described in the legend of **Figure 5**.

Lipidic exine synthesis is an important component of the pollen wall in rice and *Arabidopsis* (Yang et al., 2007). The anthers of OE142 were weakly stained by the lipid specific dye Sudan Black compared to that of Wt (Supplementary Figure S2B). Moreover, the TF gene *PTC1* was downregulated in OE142 (**Figure 5H**). Several lipid transfer proteins were also downregulated in *ptc1* anthers (Li et al., 2011). Therefore, the expression of these marker genes related to pollen sporopollenin biosynthesis was monitored by real time PCR during OE142 pollen development. Our results demonstrated that overexpression of *bHLH142* sharply reduced the expression of these genes related to sporopollenin and pollen wall biosynthesis. The transcripts of *Cyp703A3*, *Cyp704B2*, *MS2*, and *C4* were almost not detectable in OE142. The expression of *C6* was also downregulated in the anthers of OE142 at the late stage of development (**Figure 8**). Our analyses with rice *tdr* and *eat1* mutants also indicated that *MYB80* was downregulated at S9. Similarly, *PTC1*, a key regulator of sporopollenin biosynthesis, was significantly downregulated in the *eat1* anther (Supplementary Figure S5). Taken together, these results support the idea that both *MYB80* and *PTC1* regulate sporopollenin biosynthesis in both monocots and dicots. Thus, overexpressing *bHLH142* caused downregulation of *EAT1* at S9, which might severely inhibit *MYB80* and *PTC1* and reduce sporopollenin gene expression (**Figure 8**) and interrupt normal sporopollenin biosynthesis with defected pollen wall in OE142 transgenic lines.

### OE142 Anther Exhibits Parallel Changes in EAT1 Transcript and Protein

**FIGURE 9:**
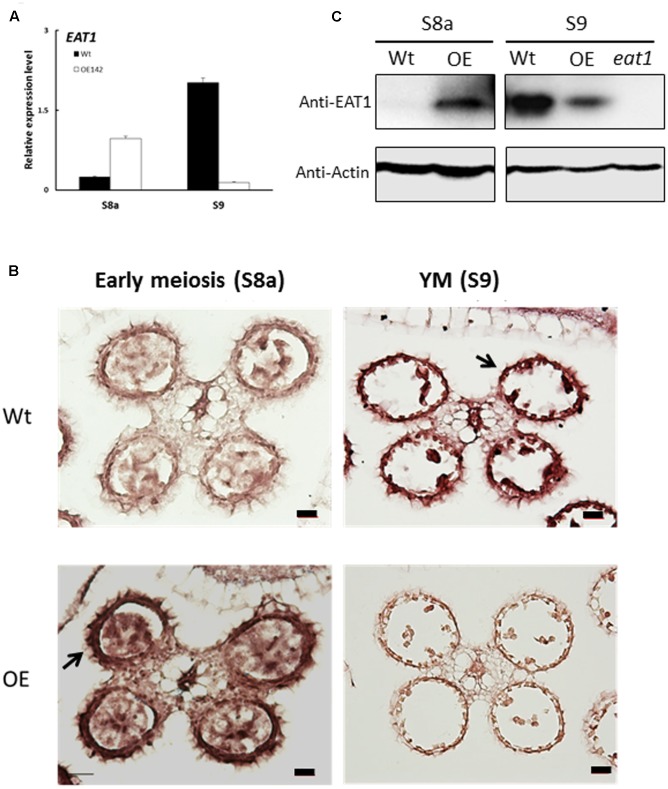
Transcript and protein levels of EAT1 were upregulated at the early stage and then downregulated at later stage of anther development in OE142. **(A)** qRT-PCR revealed upregulation of *EAT1* at (S7) and downregulation at YM (S9). **(B)** RNA ISH hybridization to *EAT1*-antisense probe in the Wt and OE142 anthers at stages S8 to S10. **(C)** Western blotting showed premature expression of EAT1 protein at MMC and downregulation at YM in OE142. The knockout mutant *eat1* was used as a negative control. Abbreviations are as described in the legend of **Figure 5**. Scale bars: 20 μm.

In this study, we found that *EAT1* was upregulated at stages S6 to S8 but then downregulated at S9 in the anthers of OE142 (**Figures 5F**, **9A**). To understand the spatial and temporal expression patterns of *EAT1* in OE142, we carried out RNA ISH hybridized *EAT1* Dig-labeling probe in the anthers of Wt vs. OE142 at S8a and S9. The results revealed that *EAT1* mRNA was highly expressed in the tapetum, middle layer, meiocyte, microspore, vascular bundle, and hull of the Wt at S9 (**Figure 9B**). However, ISH positive signal of *EAT1* was strong in the anthers of OE142 at early meiosis (S8a), but significantly reduced to a negligible level at YM (S9). Relevant tissues hybridized to *EAT1*-sense probe showed low background (Supplementary Figure S6). The ISH results support our *EAT1* qRT-PCR data (**Figure 5F**), providing a clear picture of the *in vivo* transcriptional map of *EAT1*. Western blot analysis further revealed that bHLH142 protein was specifically accumulated in the OE142 anthers (**Figure 3E**). EAT1 protein was not detectable at S8a but accumulated at a high level at S9 in the Wt (S9). However, OE142 anthers precociously expressed EAT1 protein at S8a but its expression was reduced at S9 (**Figure 9C**). Taken together, these data suggest that overexpression of *bHLH142* prematurely upregulate *EAT1* transcription (**Figures 5F**, **9A**) as well as its protein level (**Figure 9C**) in the anthers of OE142. Thus, the decreased transcript abundance and protein amount of EAT1 at S9 might interrupt the normal anther development in the OE142 transgenic lines.

In our previous study, we demonstrated that bHLH142 interacts with TDR to co-modulate *EAT1* transcriptional activity (Ko et al., 2014). Overexpression of *bHLH142* increases bHLH142 protein level, which may in turn enhance bHLH142/TDR protein–protein interaction to increase *EAT1* expression at the early stage of anther development. However, downregulation of *TDR* expression at S8 and onward (**Figure 5E**) might decrease TDR protein translation and hamper TDR/bHLH142 protein–protein interaction; therefore significantly reduce *EAT1* expression at the later stages despite the presence of high level of bHLH142 protein in the anthers of OE142. In addition, a low EAT1 protein level at S9 (**Figure 9C**) might further reduce TDR/EAT1 interaction and subsequently influence the regulatory cascade of downstream target genes and result in defected pollen development in OE142 anthers.

## Discussion

### Overexpression of bHLH142 Causes Male Sterility by Triggering Premature PCD

In an effort to provide greater insight into the functionality of bHLH142 in rice pollen development, we generated transgenic lines constitutively overexpressing *bHLH142*. To our surprise, overexpression of *bHLH142* also leads to male sterility in rice, similar to the knockout mutant reported previously (Ko et al., 2014). Except for the defect in pollen development, OE142 transgenic lines maintain Wt-like vegetative growth (**Figure 1** and Supplementary Figures S1, S2). Our results are consistent with the recent observation by Ranjan et al. (2017) who reported that overexpression of IR64 *bHLH142* in Pusa Basmati 1, as driven by maize ubiquitin promoter also causes indehiscent anthers and pollen abortion in rice. However, in our study we conducted in-depth characterization of the anther development in OE142 to elucidate the molecular events altered by overexpression of bHLH142 and employed related rice mutant plants to establish the hierarchy of the pollen development regulatory gene network. Thus, this study advances our understanding of the central role played by bHLH142 in pollen development.

Our in-depth molecular characterization suggests that overexpression of *bHLH142* significantly alters *in vivo* homeostasis of the known key pollen development-related regulatory TFs in the OE142 anthers (**Figure 5**). The key regulators of anther development, such as *UDT1*, *GAMYB*, *MYB35*, *TDR*, and *EAT1* were upregulated in OE142 at the early stages of anther development (**Figure 5**). Clearly, overexpression of bHLH142 triggers a premature onset of tapetal PCD in OE142 anthers before the maturation of pollen grains, as shown in the TUNEL assay (**Figure 2**). The reduced expression of *EAT1* at the young microspore stage (S9) and onward in OE142 anthers (**Figure 5F**) further decreased the expression of the downstream genes in the PCD pathway, such as *AP37*, *AP25*, and *CP1* (**Figure 6**) and pollen wall biosynthesis, such as *MYB80*, *PTC1*, *MS2*, *Cyp704B2*, *C4*, and *C6* (**Figure 8**). Obviously, interference with the timely and strength of expression in these pollen development associated genes leads to male sterility in OE142.

To address the underlying molecular basis responsible for the resulting phenotype by overexpressing bHLH142, Ranjan et al. (2017) used RNA-seq to profile gene expression in the Wt and OE anthers. A total of 827 genes at the tetrad stage and 735 genes at the mature pollen stage were found differentially expressed in their overexpression line. It is suggested that bHLH142 might regulate carbohydrate and lipid metabolism, cell wall modification, reactive oxygen species (ROS) homeostasis and cell death-related genes during rice anther development. These results are in accordance with our observations, but do not contribute to our understanding of the underlying role of bHLH142 in the regulatory network of anther/pollen development. This may be attributed to the low levels of expression of bHLH142 and the stage of anthers used for transcriptome analysis in their study. *bHLH142* was upregulated only 2.33-fold at the tetrad stage and 3.69-fold at the mature pollen stage (Ranjan et al., 2017, their Supplementary Tables S2, S3). However, in our study the OE142 line #96, which exhibited the highest *bHLH142* expression level (51-fold, **Figure 3A**), was used for in depth analysis. The strong overexpression of *bHLH142* in our OE142 line enabled us to detect more significant changes in differentially expressed genes, especially those involved in transcriptional regulatory network. We found significant upregulation of *UDT1*, *GAMYB*, *TDR*, and *EAT1* at the early stage in OE142 anthers. In contrast, no alternation in expression of these master regulators was detected in the RNA-seq data presented by Ranjan et al. (2017). Another potential shortfall of their study is the pollen development stages used for transcriptomic profiling. For example, the second RNA sample was collected at the mature pollen stage, which might be too late to detect any changes in TFs that control the early stage of pollen development, such as *UDT1* and *GAMYB*, etc. On the other hand, the first RNA sample taken at the meiosis stage might be too early to observe any altered expression of downstream functional genes that control lipid biosynthesis and PCD. Our qRT-PCR analyses clearly detected the alternation in expression of all major marker genes using RNAs collected from various anther developmental stages and provided more valuable information (**Figures 5**–**8**).

Although Ranjan et al. (2017, their Table 1) presented a list of some known markers of anther development that showed altered expression in their OE line, they were not verified by qRT-PCR analysis. For example, the known pollen wall synthesis marker genes such as *OsC4*, *LTP45*, *CYP703A3*, and *C6* were upregulated but *CYP704B2* was downregulated in their OE line (Ranjan et al., 2017, their Table 1). This is contradictory, as it has been clearly shown that in the pollen development regulatory network *bHLH142* is located upstream of *Cyp703A3*, *Cyp704B2*, *MS2*, and *C6* and they are involved in the same sporopollenin biosynthesis pathway (Aya et al., 2011; Ko et al., 2014). One would expect that the alternation in gene expression in the same pathway or network should be consistently up- or downregulated. Our analysis showed sporopollenin biosynthesis-related genes were all downregulated in OE142 (**Figure 8**) mainly due to the decreased expression of *EAT1* and *PTC1* at the YM stage (**Figure 5**). Consistently, the pollen of our OE line also showed weak Sudan Black staining (Supplementary Figure S2B) further suggests the low lipid content in the OE142 transgenic line.

### Tightly Regulated bHLH TFs Are Essential for Pollen Development

Our study revealed that overexpression of *bHLH142* causes significant changes in the expression of known regulatory genes associated with tapetal PCD, ROS metabolism, and pollen wall development (**Figures 5**–**8**), thus leading to male sterility in OE142 transgenic plants. Our study provided a detailed characterization of the molecular consequences of overexpressing *bHLH142* on the key regulators of pollen development that leads to male sterility. It is interesting to find that upregulation of EAT1 at the early stage of pollen development (**Figures 5F**, **9**) also promoted premature PCD in the OE142 anthers, while its downregulation at the late stage impaired pollen development by suppressing genes involved in pollen wall biosynthesis, ROS scavenging and PCD. Taken together, these events led to male sterility in OE142. This may result from the interference in its protein interaction with TDR in activational transcription of *EAT1* (Ko et al., 2014). Thus, this study advances our knowledge of the molecular mechanism underlying the bHLH142 and EAT1 transcriptional circuits controlling pollen development in rice and possibly in other plants as well. Timely expression and maintenance of proper expression levels of these bHLH TFs must be tightly regulated developmentally for normal pollen maturation. Using gain-of-function (OE142) and loss-of-function mutants (*ms142*, *tdr*, and *eat1*), the hierarchy of several known regulatory network genes associated with pollen development is therefore clarified in this study.

**FIGURE 10:**
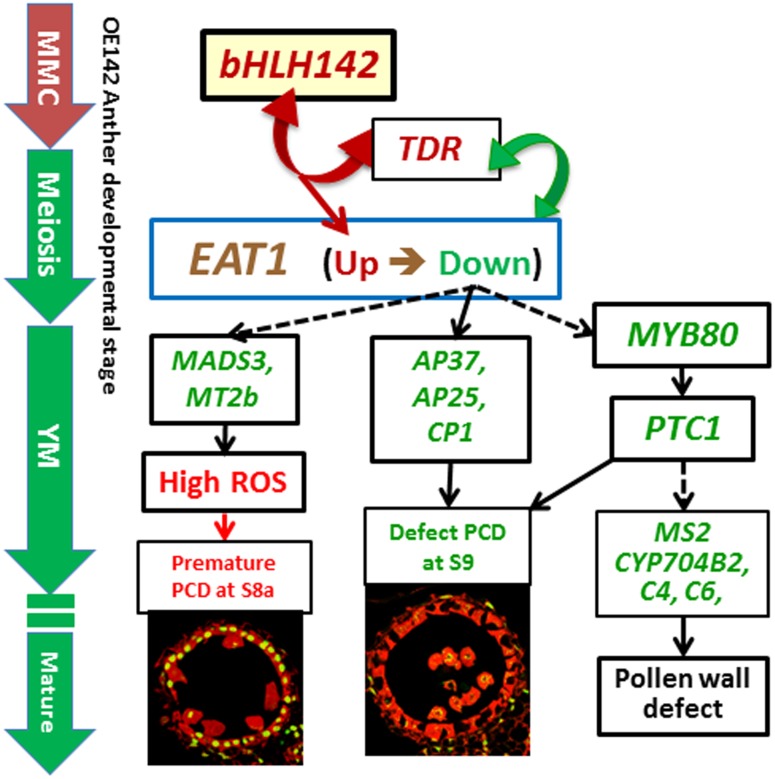
Proposed mechanistic model of male sterility in rice caused by overexpressing *bHLH142*. Overexpression of *bHLH142* upregulates *bHLH142, TDR*, and *EAT1* at an early stage of anther development cause premature onset of tapetal PCD at meiosis-I (S8a). However, *EAT1* is downregulated at the young microspore stage (YM, S9), which in turn reduces the expression of the downstream genes involved in ROS scavenging (*MADS3*, *MT2b*). Moreover, OE142 downregulated PCD marker genes (*AP37*, *AP25*, *CP1*), and sporopollenin biosynthesis genes (*MYB80*, *PTC1*, *MS2*, *CYP704B2*, *C4*, *C6*). Thus, defected tapetal PCD at YM stage (S9) and defected pollen wall development together leads to male sterility in the overexpression line. Genes marked in red or green denote upregulation or downregulation, respectively. Solid arrow lines indicate direct regulation, while dotted arrow lines indicate indirect regulation. Double arrows represent protein–protein interaction. MMC, microspore mother cell; YM, young microspore stage (S9).

Based on this and previous studies, we propose a mechanistic model of genic male sterility in rice as caused by overexpressing *bHLH142* (**Figure 10**). According to the model, overexpressing *bHLH142* causes upregulation of *bHLH142*, *TDR*, and *EAT1* at the early stage of anther development. This consequently leads to premature onset of tapetal PCD. However, *EAT1* is downregulated at the young microspore stage (YM, S9) in OE142 anthers, which in turn further reduces the expression of the downstream functional genes involved in PCD (*AP37*, *AP25*, and *CP1*), ROS scavenging (*MADS3* and *MT2b*), and pollen wall biosynthesis (*MYB80*, *PTC1*, *Cyp704B2*, *MS2*, and *C4*) and impairs normal pollen grain maturation. Thus, increased ROS accumulation, defect in timely tapetal PCD at the YM stage, and defect in pollen wall development, eventually lead to male sterility in the OE142 plants. The alterations in homeostasis of key TFs in pollen development or protein–protein interaction between bHLH142/TDR or TDR/EAT1 may account for the decreased expression of downstream pollen development marker genes regulated by EAT1 (Ko et al., 2014).

### Potential of Establishing a Male Sterility Line by Overexpressing Key TFs

Our finding that overexpression of *bHLH142* (*TIP2*) causes male sterility by triggering premature PCD in rice is similar to previous results obtained by overexpressing several pollen development related TFs in other species. A total of 148 out of 196 *Arabidopsis* transformants overexpressing *AMS* (rice homolog of *TDR*) produced sterile pollen, mimicking the *ams* mutant phenotype. It has been claimed that the resulting male sterility might be due to co-suppression of *AMS* (Sorensen et al., 2003). Moreover, overexpressing *MS1* as driven by the CaMV35S promoter also caused stunted plants with sterile pollen in *Arabidopsis* (Yang et al., 2007). Recently, the ortholog of *MS1* in barley (*HvMS1*) was cloned and its expression was altered to be either overexpressed or suppressed, both RNAi and overexpression of *HvMS1* full-length cDNA under the control of the maize ubiquitin promoter caused male sterile phenotype in the transgenic barley plants (Fernandez Gomez and Wilson, 2014). Also, knockout of *AtCEP1*, which encodes a papain-like cysteine protease involved in tapetal PCD, delayed tapetal PCD, while its overexpression caused premature tapetal PCD (Zhang et al., 2014). This work tends to suggest that altered homeostasis of the related TFs changed in pollen regulatory cascade may be the major cause. Thus, we hypothesize that overexpression of other key TFs in the pollen development regulatory network, such as *GAMYB*, *UDT1*, *TDR*, or *EAT1* (*DTD*, *bHLH141*), may also cause male sterile phenotype in rice due to alteration in the dynamic of the gene regulatory cascades in pollen development.

### Advantages of Using OE142 in Hybrid Seed Production

In this study, overexpression of *bHLH142*, an anther-specific TF gene, by a strong constitutive promoter led to its ubiquitous transcription in leaves, hulls, as well as in the anther of OE142, as expected (**Figure 3**). However, bHLH142 protein expression was not constitutively expressed. Rather, its expression was maintained in a tissue specific manner, as in the Wt anthers (**Figures 3E**, **4**). The ubiquitin promoter is expected to drive ubiquitous gene expression. However, protein expression level is determined by the rate of transcription and by post-transcriptional processes that lead to changes in mRNA transport, stability, and translational efficiency. In fact, overexpressing target genes in an anther-specific manner is desirable from the perspective of GMO food biosafety because anther-specific expression will avoid any unintended expression of transgenes in other tissues, especially in the edible part of the seed (**Figure 4**). This type of genetic modification may be deemed more acceptable by consumers. Here, we showed that overexpression of *bHLH142* may provide a novel and simple way to generate genic male sterility lines in rice. Moreover, genetic engineering using the overexpression approach is preferred to RNAi by the biotech industry.

## Author Contributions

S-SK designed the experiments; M-JL performed most of the experiments; Y-JL, H-XH, T-TY, T-KC, C-MJ, and S-SK conducted parts of the experiments; M-JL and S-SK analyzed and prepared the data; S-SK and MK wrote the manuscript.

## Conflict of Interest Statement

The authors declare that the research was conducted in the absence of any commercial or financial relationships that could be construed as a potential conflict of interest.
