# Supplementary material for: Tightly Controlled Expression of bHLH142 Is Essential for Timely Tapetal Programmed Cell Death and Pollen Development in Rice
Source: Front Plant Sci. 2017 Jul 18;8:1258. doi: 10.3389/fpls.2017.01258 (PMC5513933; doi:10.3389/fpls.2017.01258)
Supplement: Supplementary file 1 [file Image_1.PDF]

Table S1. Primers used in this work.

Primer Name	RAP Accession No.	Sequence (5’ →3’)
**Gene construction**		
S80qPCR-F3_BamHI	Os01g0293100	CGGGATCCGGTGGTAGAGTGCGAGGAAGATGT
S80FLcds-R2_BamHI		GGATC CTGCA AACCA TGACA TACCA AAGAT C
S80cDNA-BamHI-F	Os01g0293100	AGGGATCCTGGTAGAGTGCGAGGAAG
S80cDNA-XbaI-R		GCTCTAGAGTACTCATCCACCACTTC
**Screening of transgenic line**		
Hyg-F		GATGTAGGAGGGCGTGGATA
Hyg-R		CGTCT GCTGC TCCAT ACAAG
**Quantitative/ RT- PCR**		
S80qPCR-F3	Os01g0293100	GGAGCACGTACATCCAGCGG
S80-GT-R3		ACTCATCCACCACTTCAATCAGCC
GAMyb-F	Os01g0812000	CATCCTGGTCCATTCCTCAATGAC
GAMyb-R		TTCAGGATGAGGTGAAGTGTCCC
UDT1-F	Os07g0549600	GATCTTCTGGACCAAGAGGGCAG
UDT1-R		GTCAGGAGTGTCTCAGATGCTTGG
OsTDR-F	Os02g0120500	CGCTCGCTCGTCCCAAACAT
OsTDR-R		CGGTCATTGCTGGGTCCTTGT
bHLH141-F	Os04g0599300	TGGTGGAACAGAAGAGGCATGG
bHLH141-R		GCATGAAGCAGAGAGTTGGCCTT
OsPTC1-F	Os09g0449000	GACCTCTGGGACCGCATTTG
OsPTC1-R		TGAAGCACGACAGGTGCGG
AP37-F	Os04g0448500	AGGCGGGCAGCGTCTCCAT
AP37-R		CCATAAGCCAGCCACGATGATGA
AP25-F	Os03g0186900	CCGCAGAACGTCAACTCCGT
AP25-R		GCACAAGCCGTTCTCGTGC
OsCP1-F	Os09g0381400	GGACCACCTGCTGCTGCAACT
OsCP1-R		GAACACTTCGTGCCATCGCC
CYP703A3-F	Os08g0131100	GGCGTCATCCTGGTGCTCAT
CYP703A3-R		CGACGCCTAGCTCAAACTTGC
OsMS2-qRT-F	Os03g0167600	TGGAGCAGTTCGCCAGCTACG
OsMS2-qRT-R		CTTCTCCTCCTCCGACATCTCCC
CYP704A2-F	Os03g0168600	GGTTGAGATCGGGACGCTGT

CYP704A2-R		ATCACCTTCTCTTGCTTGCCG
OsC6-RT-F	Os11g0582500	GGGCTGTCGTCCATCGTGA
OsC6-RT-R		CGCAGGTGGAGATGATGTCC
OsUBQ5-F	Os01g0328400	GCGGAAGTAAGGAAGGAGGAGG
OsUBQ5-R		GGCATCACAATCTTCACAGAGGTG
MADS3-F	Os01g0201700	GCAGTAATTCTATGTGTTCGTCAGATC
MADS3-R		GATCAGTTACACGAATCAGAGCACA
MT2b-F	Os05g0111300	GCTGCTCCTGCTGCTCCTG
MT2b-R		ACTGACAACGACGACGACGG
OsMYB80-F	Os04g0470600	CATGAGCGATCCCTCCACCG
OsMYB80-R		CTGGTGGCTCCACATCGACGT
OsC4-F	Os08g0546300	CTGCCTAAGACGAGACGAGAGT
OsC4-R		GCCAAAGGAGGTCATCGTTAA
***In-situ* hybridization analysis**		
S80qPCR-F2	Os01g0293100	CATGTTCAACACCAAGATTCATTCG
S80FLcds-R2		TGCAAACCATGACATACCAAAGATC
bHLH141-F	Os04g0599300	TGGTGGAACAGAAGAGGCATGG
bHLH141-R		GCATGAAGCAGAGAGTTGGCCTT

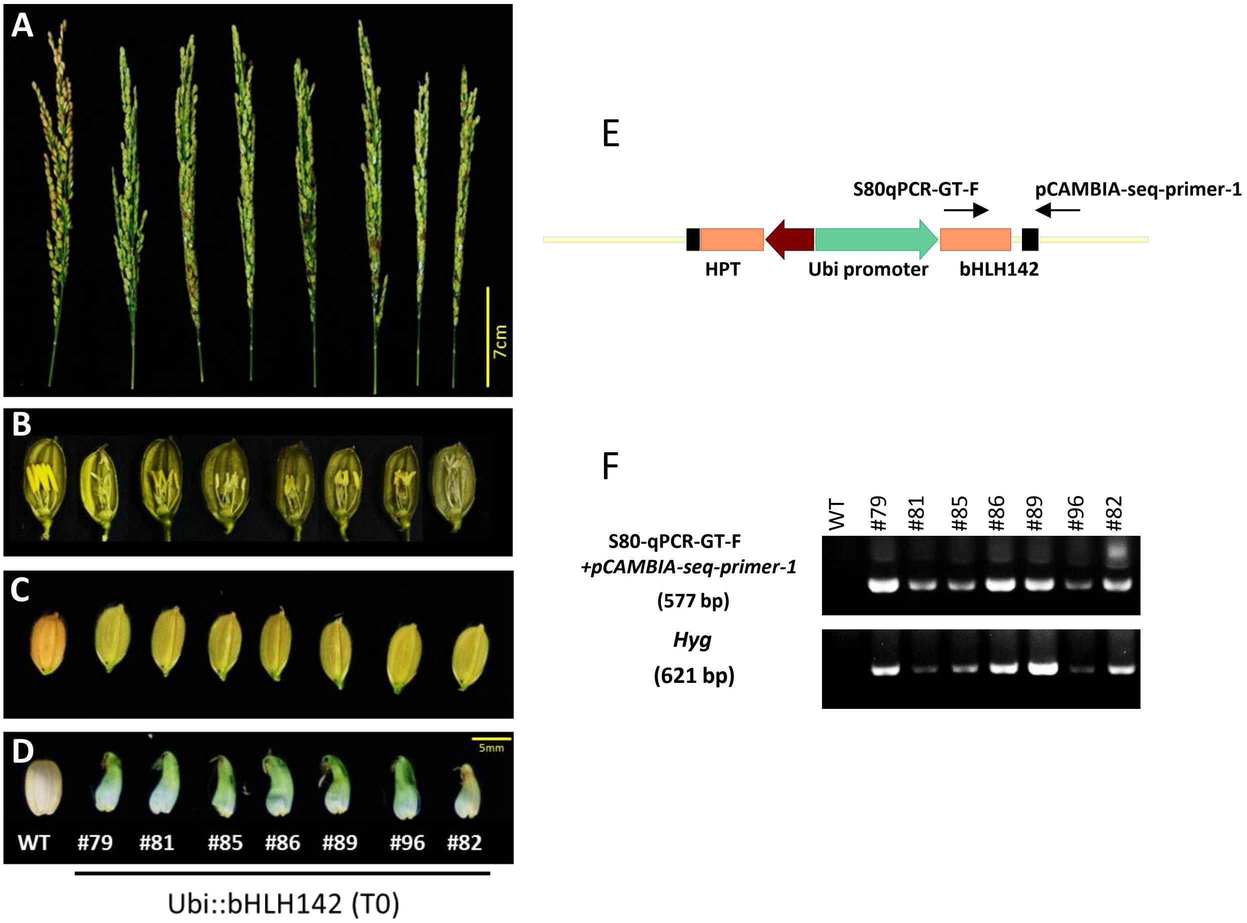

Supplementary Fig. S1. ***bHLH142*** **overexpressing transgenic lines.**
(A) Panicles of wild-type (Wt) and different OE142 T_0 lines at the mature stage. (B) Spikelets of Wt (left) and several OE142 T_0 lines at one day before anthesis. (C) Grains of Wt (filled) and several OE142 T_0 lines at the harvest stage. (D) De-hulled seeds of Wt (filled) and several OE142 T_0 lines showing inviable seeds at the harvest stage. (E) Construct map and primer design for genomic PCR to confirm T-DNA insertion. (F) Genomic PCR confirmed T-DNA insertion in different OE142 lines.

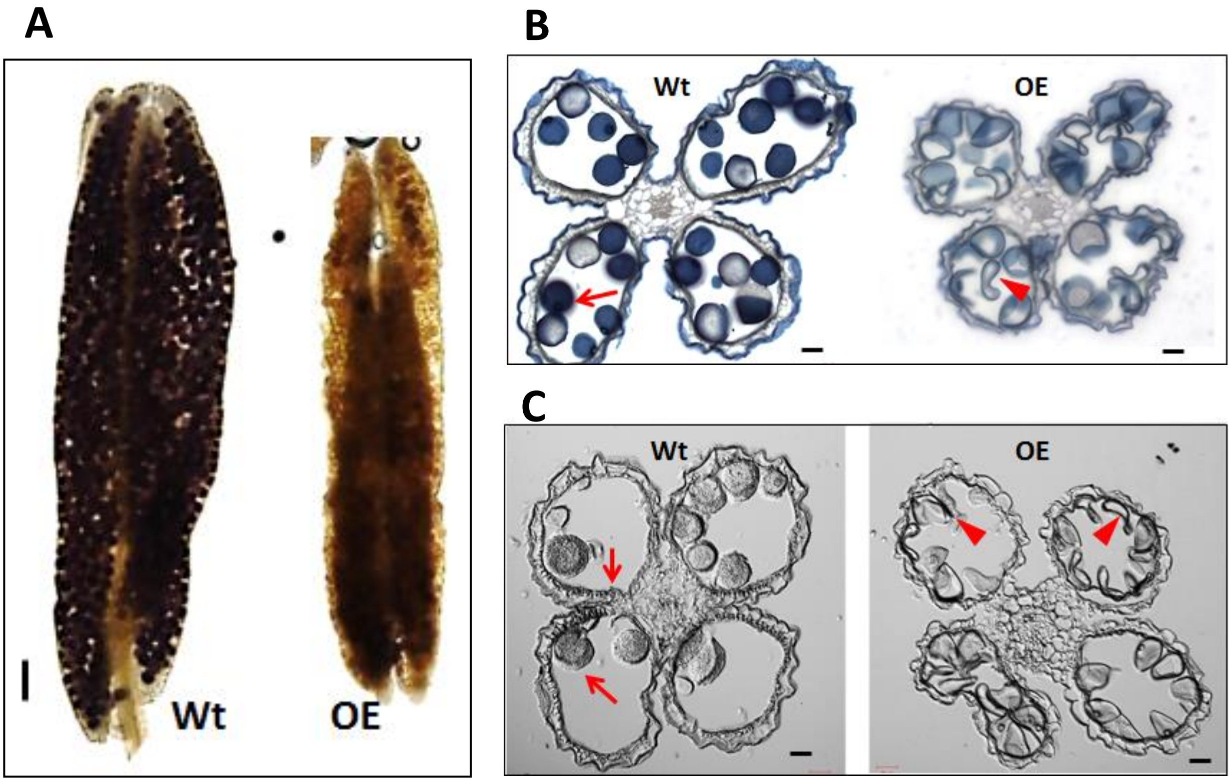

Supplementary Fig. S2. Overexpression of *bHLH142* (OE142) resulted in defect in anther development in rice.

(A) Staining of anther and pollen grains by 2% I2/KI solution in the wild-type (Wt) and OE142 line #96 at 1 day before anthesis (DBA). (**B)** Weak staining of Sudan Black in the transverse anther section of OE142 (right panel) compared to the Wt (left panel) at 1 DBA. **(C)** Transverse anatomical comparison of anther of the wild-type (Wt) and OE142 1 DBA using DIC. Arrows show the fertile pollens (B) and the thickening of endothelial cell layers, prior to anther dehiscence (**C**). Arrowheads show degenerated pollen (**C**, right panel). Scale bars: 100 um **(A)**, 20 um **(B, C)**.

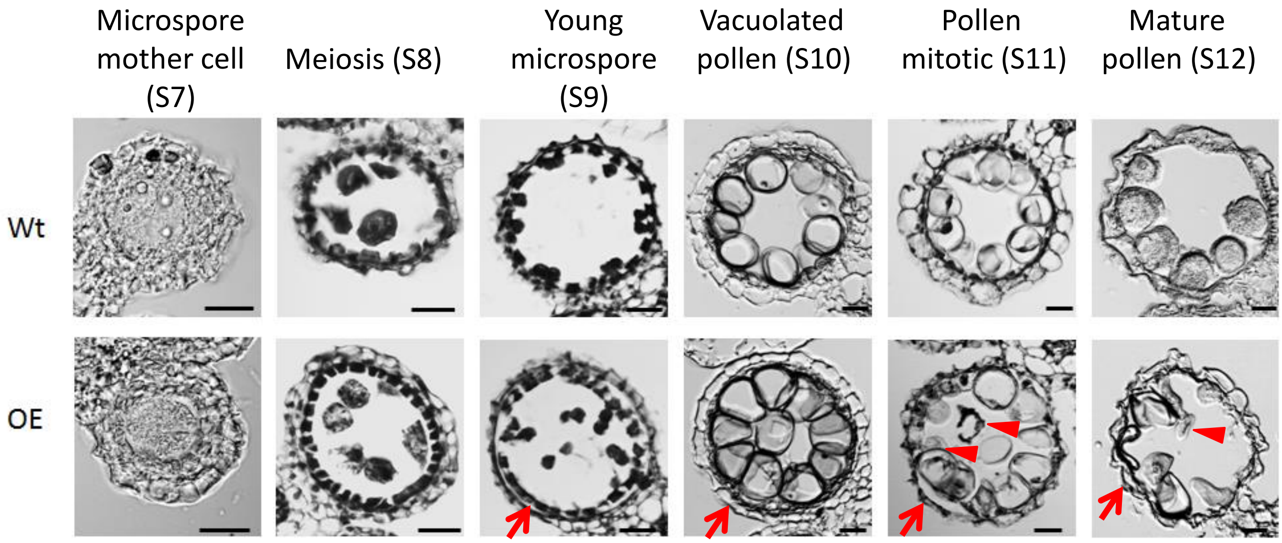

Supplementary Fig. S3. Transverse sections showing defect of anther development in OE142 line. Arrows indicate thin epidermal layer in OE142 anther. Arrowheads show the degenerated pollen. Scale bars: 20 µm.

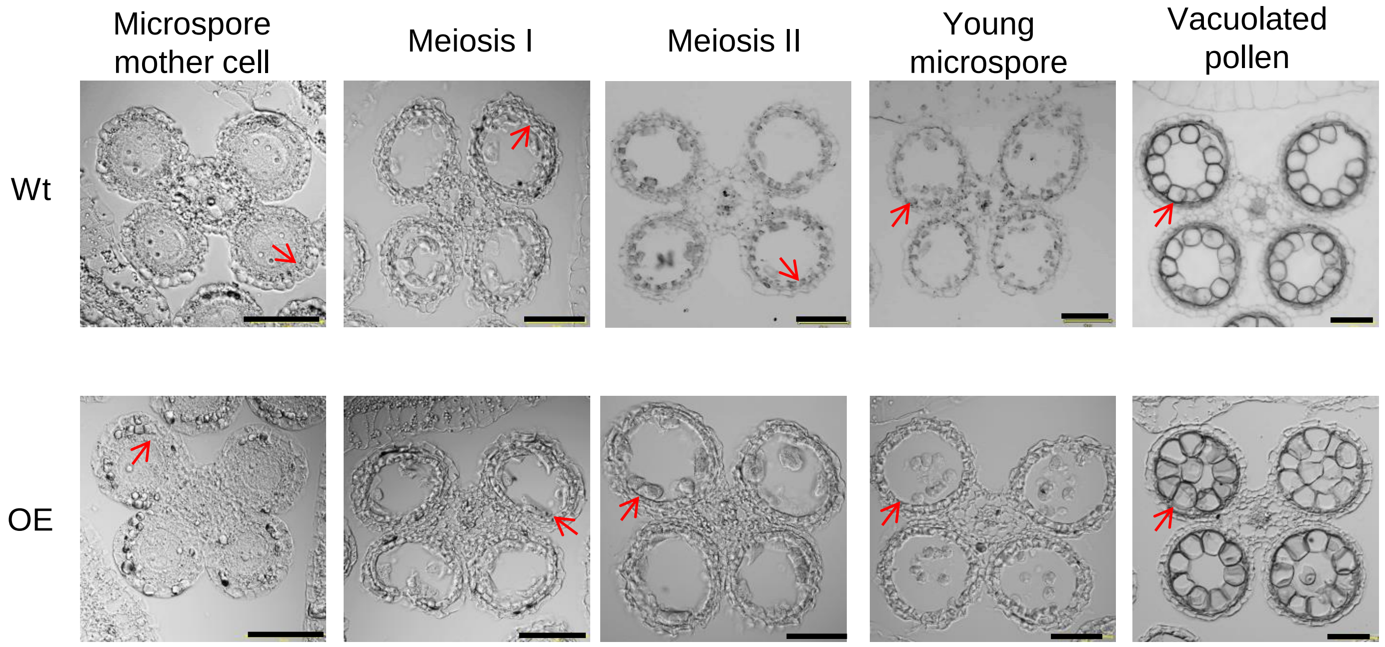

Supplementary Fig. S4. Differential interference contrast (DIC) images of anther cross sections corresponding to TUNEL assay.

Upper panel: wild type, lower panel: OE142. Arrows indicated tapetal layers. Scale bars: 50 µm.

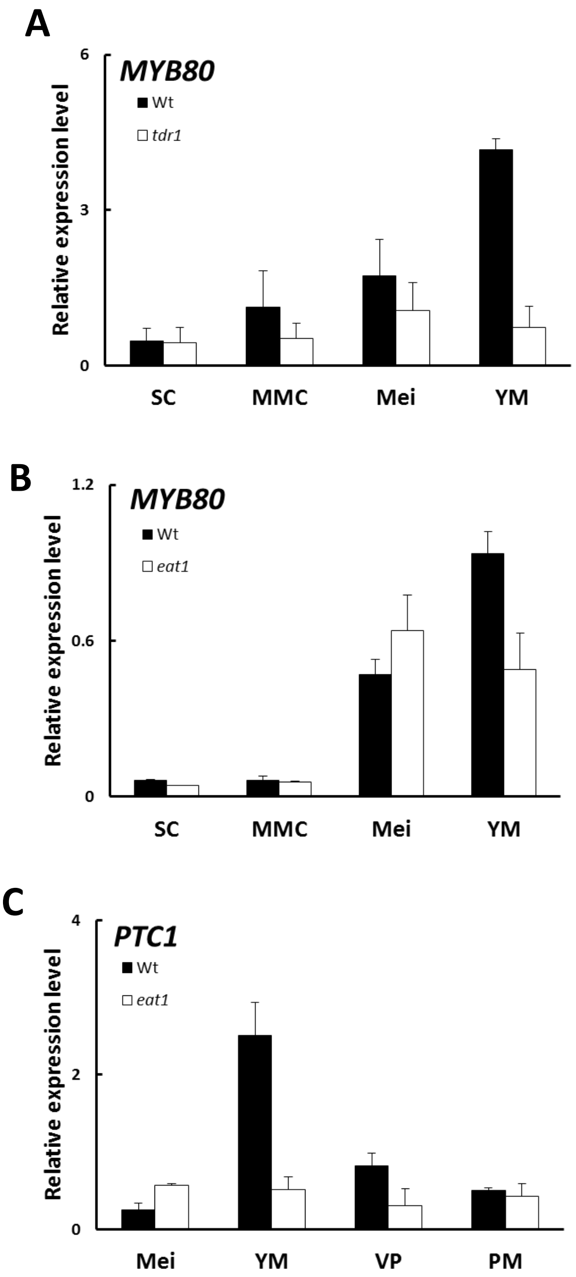

Supplementary Fig. S5. Mutagenesis analysis indicated gene hierarchy of *MYB80* and *PTC1*. Wild type for *tdr1* is Dongjin, *eat1* is in Hitomebore background. Abbreviations are as described in the legend of Fig. 5.

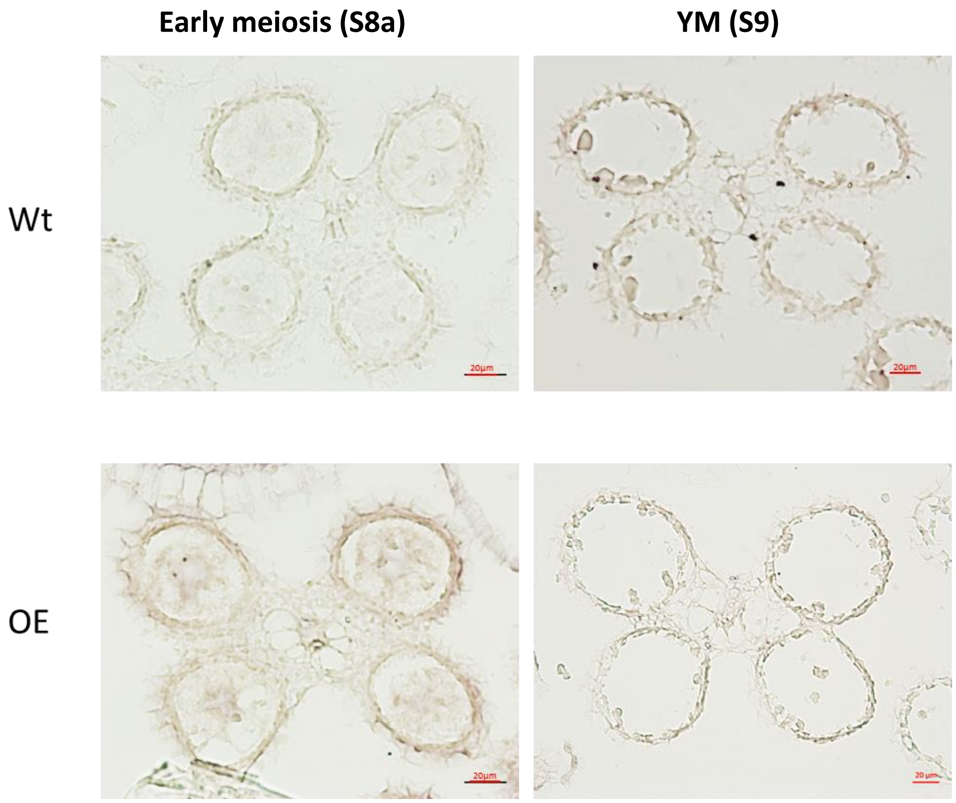

Supplementary Fig. S6. RNA *ISH* to *EAT1*- sense probe to anthers at various developmental stages of the Wt and OE142. Scale bars: 20 µm.
